# Insulin signaling in skeletal muscle during inflammation and/or immobilisation

**DOI:** 10.1186/s40635-023-00503-9

**Published:** 2023-03-27

**Authors:** Julius J. Grunow, Thomas Gan, Heidrun Lewald, J. A. Jeevendra Martyn, Manfred Blobner, Stefan J. Schaller

**Affiliations:** 1 Charité - Universitätsmedizin Berlin, corporate Member of Freie Universität Berlin and Humboldt-Universität zu Berlin, Department of Anesthesiology and Operative Intensive Care Medicine (CVK, CCM), Charitéplatz 1, 10117 Berlin, Germany; 2 Technical University of Munich, School of Medicine, Klinikum rechts der Isar, Department of Anesthesiology and Intensive Care, Ismaninger Straße 22, 81675 Munich, Bavaria Germany; 3 Department of Anaesthesia, Critical Care and Pain Medicine, Massachusetts General Hospital, Shriners Hospitals for Children®-Boston, and Harvard Medical School, 51 Blossom Street, Room 206, Boston, 02114 MA USA

**Keywords:** Intensive care unit acquired weakness, Immobilisation, Inflammation, Muscle atrophy, Insulin signaling

## Abstract

**Background:**

The decline in the downstream signal transduction pathway of anabolic hormone, insulin, could play a key role in the muscle atrophy and insulin resistance observed in patients with intensive care unit acquired weakness (ICUAW). This study investigated the impact of immobilisation via surgical knee and ankle fixation and inflammation via *Corynebacterium parvum injection*, alone and in combination, as risk factors for altering insulin transduction and, therefore, their role in ICUAW.

**Results:**

Muscle weight was significantly decreased due to immobilisation [estimated effect size (95% CI) − 0.10 g (− 0.12 to − 0.08); *p* < 0.001] or inflammation [estimated effect size (95% CI) − 0.11 g (− 0.13 to − 0.09); *p* < 0.001] with an additive effect of both combined (*p* = 0.024). pAkt was only detectable after insulin stimulation [estimated effect size (95% CI) 85.1-fold (76.2 to 94.0); *p* < 0.001] irrespective of the group and phosphorylation was not impaired by the different perturbations. Nevertheless, the phosphorylation of GSK3 observed in the control group after insulin stimulation was decreased in the immobilisation [estimated effect size (95% CI) − 40.2 (− 45.6 to − 34.8)] and inflammation [estimated effect size (95% CI) − 55.0 (− 60.4 to − 49.5)] groups. The expression of phosphorylated GS (pGS) was decreased after insulin stimulation in the control group and significantly increased in the immobilisation [estimated effect size (95% CI) 70.6-fold (58.8 to 82.4)] and inflammation [estimated effect size (95% CI) 96.7 (85.0 to 108.5)] groups.

**Conclusions:**

Both immobilisation and inflammation significantly induce insulin resistance, i.e., impair the insulin signaling pathway downstream of Akt causing insufficient GSK phosphorylation and, therefore, its activation which caused increased glycogen synthase phosphorylation, which could contribute to muscle atrophy of immobilisation and inflammation.

**Supplementary Information:**

The online version contains supplementary material available at 10.1186/s40635-023-00503-9.

## Background

In approximately 40% of Intensive Care Unit (ICU) patients an acquired Weakness (ICUAW) can be detected [1]. Depending on the constellation of risk factors, such as immobilisation, hyperglycaemia, or severity of systemic inflammation, the incidence of ICUAW diagnosis can increase to 86% [2]. ICUAW has a profound short—as well as long-term impact on patients such as delayed weaning of mechanical ventilation and/or deferred discharge from the hospital. Mortality and morbidity in ICUAW patients has been shown to be dramatically increased up to 5 years after discharge [3].

Muscle atrophy and insulin resistance are two main contributing pathophysiologic factors identified in ICUAW. These factors are highly interdependent making it difficult to investigate them separately in humans [4, 5]. The two triggers for insulin resistance that are invariably present during critical illness are inflammation and immobilisation. Mobilization on the other hand has been shown to increase insulin sensitivity in critically ill [6].

Current management recommendations for ICUAW aim at minimising immobilisation and insulin resistance [7]. Early protocol-based mobilization has been shown to improve functionality and counteract muscle atrophy as well as insulin resistance [6, 8, 9]. Nevertheless, evidence has not always been uniform and some studies failed to demonstrated the association [10]. Although controversial, intensive insulin therapy has been shown to have a protective effect regarding the development of neuromuscular dysfunction [11]. While early mobilization has been implemented in clinical routine, intensive insulin therapy is still under discussion due to multiple large scaled randomized-controlled trials showing increased mortality or serious adverse events [12].

Weber-Carstens and colleagues investigated the insulin signaling pathway in critically ill patients. They observed a significant insulin resistance using a euglycemic hyperinsulinemic clamp and at the end of the insulin signaling pathway insufficient translocation of GLUT4 transporters to the sarcolemma. Since they investigated the insulin signaling pathway up to the point of Akt phosphorylation and were not able to identify any aberrant signaling, the insulin signaling dysfunction seems to be downstream of Akt, which was not investigated in this study [5]. Contrary to these observations, in an animal burn model, phosphorylation of Akt and its ability to transduce anabolic signals downstream was significantly impaired [13]. Thus, further investigations to examine the roles of immobilisation and inflammation could point to the primary aetiologic factor leading to muscle wasting in critical illness.

Hence, this study investigated the effect of immobilisation and inflammation alone and their combination in decreasing the anabolic response to exogenous insulin by examining signaling changes downstream of Akt. The insulin transduction pathway was studied in skeletal muscle in an established rat model of immobilisation and/or inflammation, previously described by us [14].

## Methods

All experiments described in the study protocol were approved and performed according to the guidelines by governmental veterinary and animal protection office (Regierung von Oberbayern, TVA 55.2-1-54-2532-121-2015; approval date 12th of August 2015).

### Animal model and study design

**Fig. 1:**
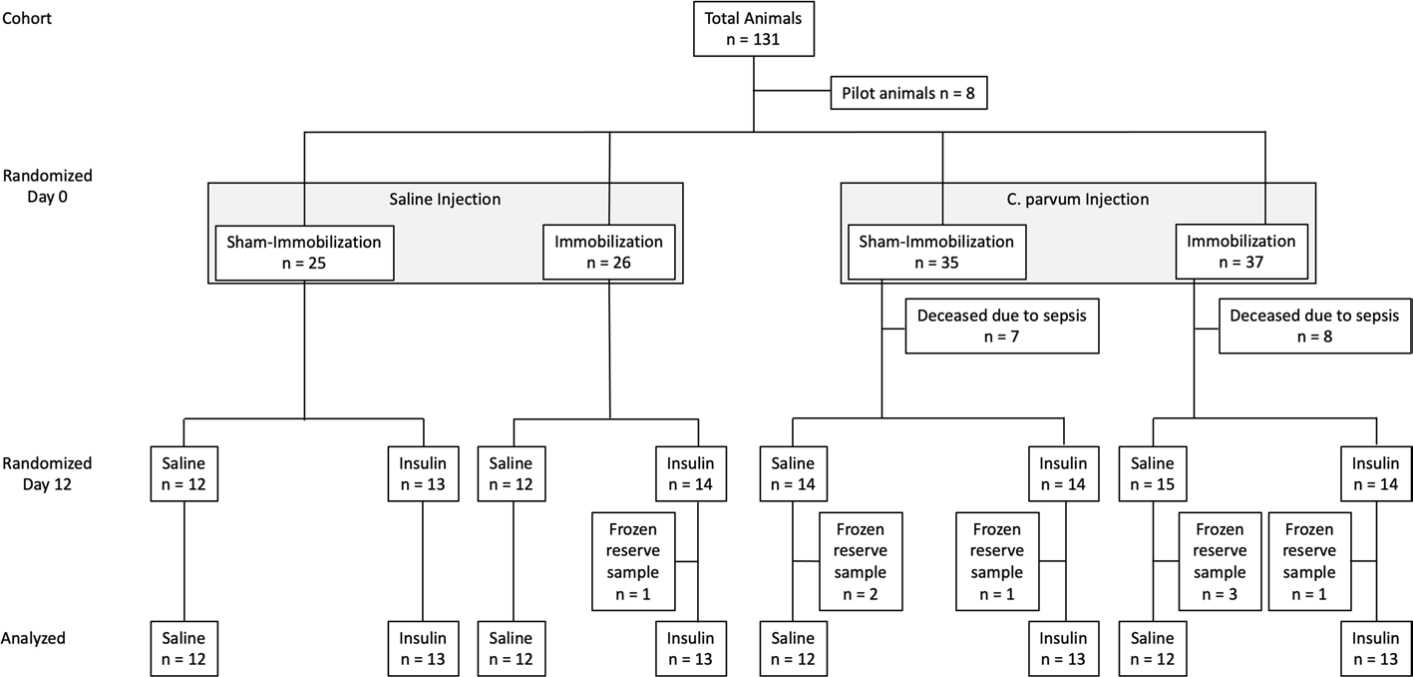
Study scheme. Single-hit and double-hit models of systemic inflammation and/or immobilisation with and without insulin

Details of the methodology are described in the appendix. In brief, male Sprague Dawley rats (Charles River, Germany) were initially (day 0) randomized within a double hit model to either control, immobilisation alone, inflammation alone or the combination of immobilisation and inflammation as described previously [14, 15]. After 12 days of perturbation, the rats were further randomized to receive either an insulin or a saline bolus injection (see Fig. 1).

#### Immobilisation

On day 0, all animals received general anaesthesia and underwent Kirschner wire immobilisation of the ankle and knee joint at 90° of one leg. In rats of “sham-immobilisation” group, wires were immediately removed to control for effects of the surgery. All animals received 0.01 mg/kg buprenorphine i.p. once to ensure sufficient analgesia and comfort without affecting the insulin signaling pathway.

#### Systemic inflammation

All animals randomly received an injection of either 56 mg/kg heat-inactivated *Corynebacterium parvum* (Hoffmann-La Roche, Switzerland) or 0.5 ml saline via the dorsal penile vein on days 0, 4, and 8. Sustained systemic inflammation was confirmed by measuring venous methaemoglobin levels via routine blood gas analysis on days 0, 4, 8, and 12 [16]. Body weight was measured at the same time points to evaluate the effect of the systemic inflammation.

On day 12, after 6–12 h of fasting, animals were secondarily randomised to receive either 0.65 IU/kg insulin or an equivalent volume of saline via laparotomic portal vein injection [13, 17]. For this procedure, animals received general anaesthesia with isoflurane. Tibialis anterior muscles were harvested 5 min after the injection and weighed to evaluate the effect of systemic inflammation on muscle mass. Animals were subsequently euthanised. Muscles were immediately snap frozen in liquid nitrogen and stored at − 80 °C until analysis later.

### Protein analysis by immunoblot

Expression of protein kinase b (Akt), phosphorylated protein kinase b (pAkt), glycogensynthase-kinase-3-beta (GSK), phosphorylated glycogensynthase-kinase-3-beta (pGSK), glycogen synthase (GS) and phosphorylated glycogen synthase (pGS) were assessed semiquantitatively using the Western blot technique. The primary antibodies were used in a 1:5,000 dilution and the secondary antibodies in a 1:10,000 dilution (see Additional file 1: Table S1). Bands were visualized with the Bio-Rad Molecular Imager® ChemiDoc™ XRS + (Bio-Rad Laboratories, Inc., USA) and analysed with ImageLab (Bio-Rad Laboratories, Inc., USA). Expression values were normalised to total protein [18].

### Statistical analysis

Data are shown as mean and standard deviation for continuous variables as well as count and percentage for categorical variables. Relative amount of phosphorylated protein was determined as the quotient between total amount of phosphorylated protein and non-phosphorylated protein within the same group. A linear mixed model was applied to analyse the main effects of immobilisation, inflammation, side and insulin treatment on methaemoglobin concentrations, body weight, muscle weight or protein concentration of operated/contralateral legs. If the main factor (i.e., immobilisation, inflammation, side, and insulin) proved to be significant, post-hoc analyses were then performed to investigate differences between or within the experimental groups. Effect sizes are reported as estimated marginal means. Significance level was set at *p* < 0.05. Statistical analysis was performed with SPSS Statistics 27 (IBM, USA).

## Results

In total, 131 rats were included into the experiment. Eight animals were used for establishing the experimental procedures in the laboratory, so that 123 animals were randomized into the four groups. 15 animals with systemic inflammation (8 with and 7 without concomitant immobilisation) had a rapidly deteriorating general condition and had to be euthanised as per the approved protocol. Therefore, 108 animals were available for the final analysis: 12 samples per group in the saline groups and 13 in the insulin groups (*n* = 100) were analysed, resulting in at least 10 analysable western blot samples per group and leg side. A total of 8 samples were kept as frozen reserve samples (see Fig. 1).

### Methaemoglobin (Meth-Hb)

**Fig. 2:**
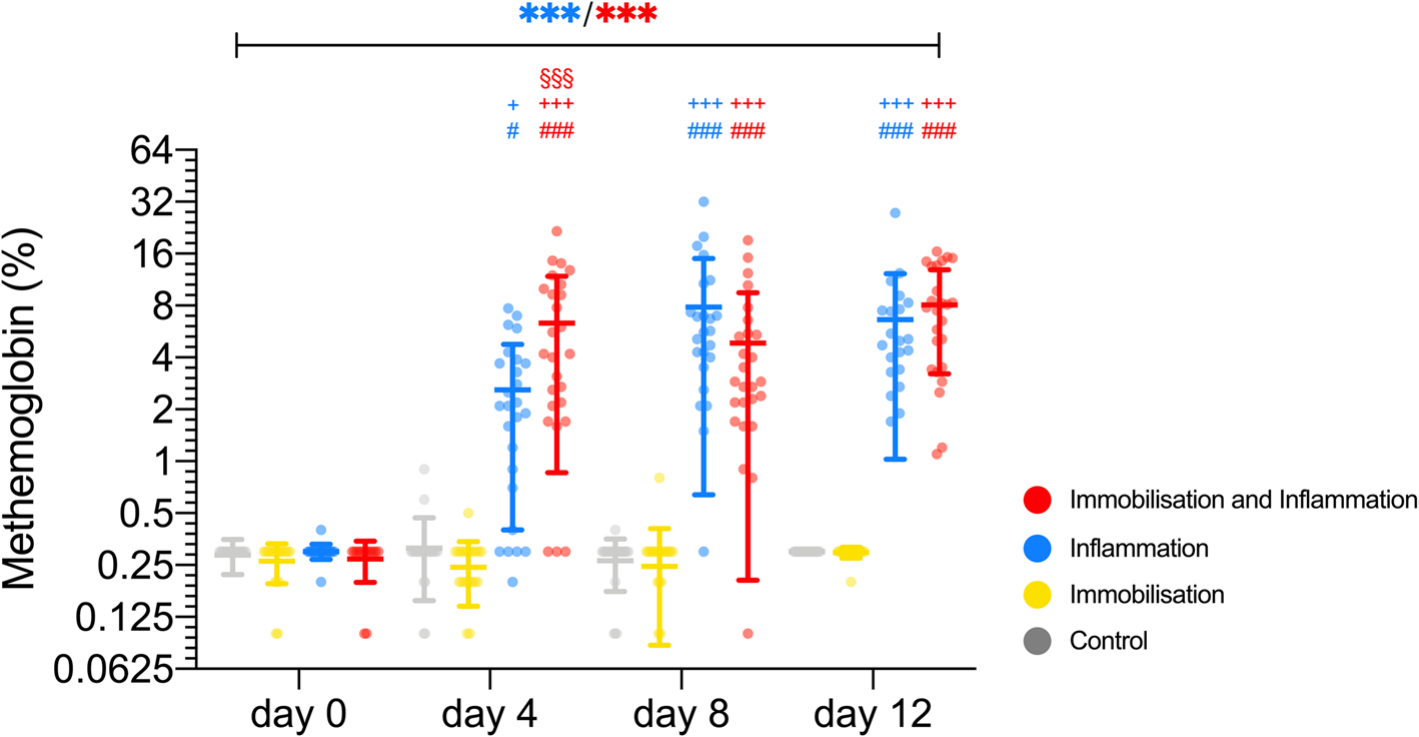
Methaemoglobin (MetHb) blood levels in %. The control (mean [SD]; day 0: 0.29 [0.06], day 4: 0.31 [0.16], day 8: 0.26 [0.09], day 12: 0.30 [0.00]; *p* = 0.380) and immobilisation (mean [SD]; day 0: 0.26 [0.08], day 4: 0.25 [0.09], day 8: 0.25 [0.16], day 12: 0.30 [0.02]; *p* = 0.293) group showed no change in methaemoglobin blood levels over time while in the inflammation (mean [SD]; day 0: 0.28 [0.08], day 4: 2.65 [2.31], day 8: 7.07 [5.73], day 12: 6.50 [5.09]; *p* < 0.001) as well as immobilisation and inflammation (mean [SD]; day 0: 0.28 [0.05], day 4: 6.22 [5.57], day 8: 4.77 [4.62], day 12: 8.06 [4.75]; *p* < 0.001) groups a significant increase over time was present. This led to significantly increased values in the inflammation group in comparison with the control and immobilisation group on day 4 (*p* = 0.035/p = 0.027) as well as on day 8 (*p* < 0.001 for both groups) and 12 (*p* < 0.001 for both groups). The immobilisation and inflammation group showed an increase over all three other groups on day 4 (*p* < 0.001 for all three groups) and over the control and immobilisation groups on day 8 (*p* < 0.001 for both groups) and 12 (*p* < 0.001 for both groups). The scale of the y-axis has been Log2 transformed. The number of identical symbols at the top of the error bars indicate the significance level: one < 0.05; two < 0.01 and three < 0.001. *showed significant difference over time with the colour of the symbol indicating the respective group. ^#^ showed significant difference to the control group at the specific time point. ^+^Significant difference to the immobilisation group at the specific time point. § showed significant difference to the inflammation group at the specific time

Met-Hb as a surrogate parameter for inflammation showed an increase over time only after injection of *Corynebacterium parvum* (*p* < 0.001) without an interaction to immobilisation (*p* = 0.201) (Fig. 2). On day 12, the difference between animals having received *Corynebacterium parvum* injection (inflammation as well as immobilisation and inflammation) and those that did not (control and immobilisation) was estimated to be 4.2% (95% CI: 3.6 to 4.8).

### Body weight

**Fig. 3:**
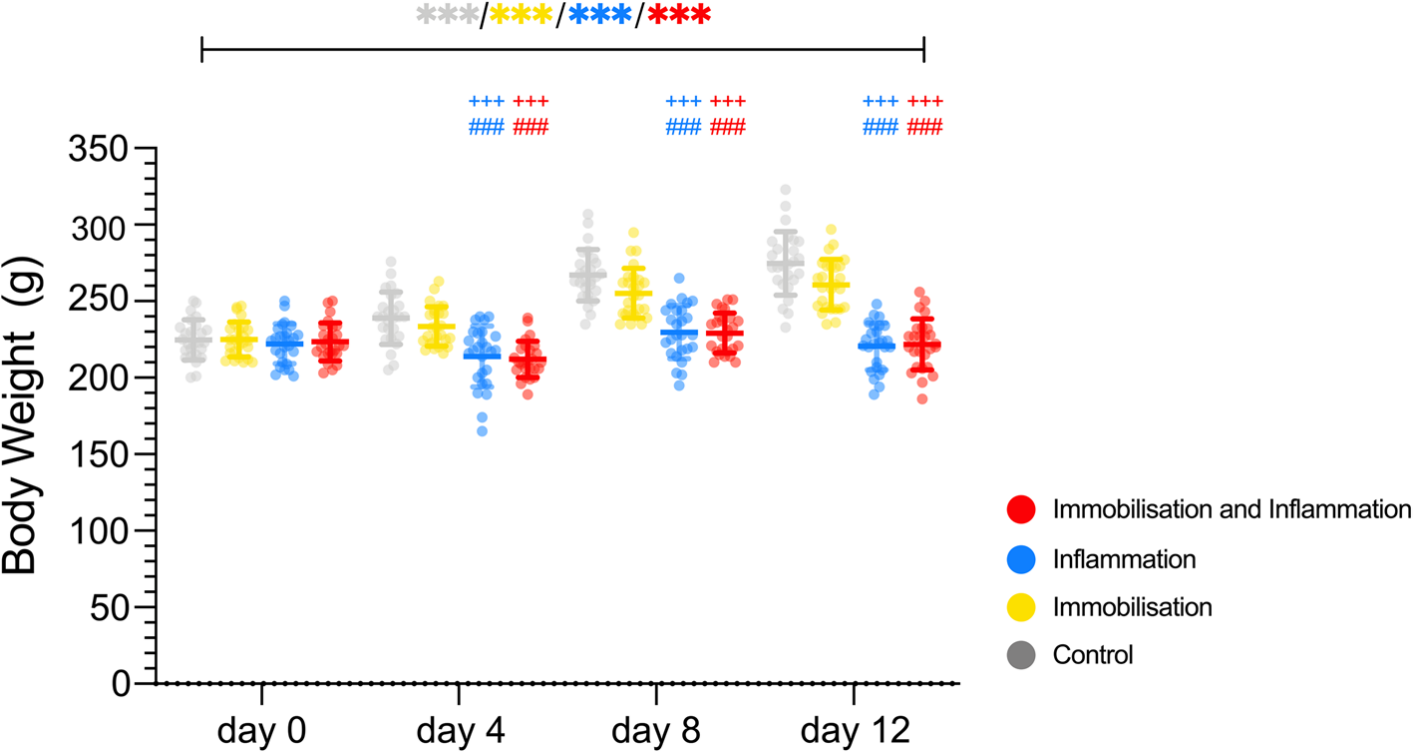
Body weight in gram. The control (mean [SD]; day 0: 224.9 [13.3], day 4: 239.5 [17.3], day 8: 267.8 [16.7], day 12: 276.0 [20.0]; *p* < 0.001), immobilisation (mean [SD]; day 0: 225.0 [11.4], day 4: 233.5 [12.9], day 8: 255.2 [16.3], day 12: 260.8 [16.5]; *p* < 0.001), inflammation (mean [SD]; day 0: 222.2 [13.0], day 4: 214.1 [19.5], day 8: 229.8 [17.5], day 12: 220.6 [15.3]; *p* < 0.001) as well as immobilisation and inflammation (mean [SD]; day 0: 223.8 [12.8], day 4: 213.0 [13.6], day 8: 230.0 [14.4], day 12: 221.2 [15.9]; *p* < 0.001) group showed a significant change over time. The increase over time in the control and immobilisation group led to significantly higher values in comparison with either the inflammation or immobilisation and inflammation group on days 4, 8 and 12 (*p* < 0.001 for all three timepoints and 3 comparisons). The number of identical symbols at the top of the error bars indicate the significance level: one < 0.05; two < 0.01 and three < 0.001. *Significant difference over time with the colour of the symbol indicating the respective group. ^#^Significant difference to the control group at the specific time point. ^+^Significant difference to the immobilisation group at the specific time point. ^§^Significant difference to the inflammation group at the specific time point

Body weight on day 0, before perturbations was 224.0 [12.5] g. There was a significant main effect for time (*p* < 0.001) and inflammation (*p* < 0.001) with a significant interaction of time with inflammation (*p* < 0.001). The inflammation (220.6 [15.3] g; *p* < 0.001 for both groups) and immobilisation superimposed on inflammation (221.2 [15.9] g; *p* < 0.001 for both groups) group showed a significantly decreased body weight compared to the control (276.0 [20.0] g) as well as immobilisation (260.8 [16.5] g) group on day 12 (Fig. 3). The body weight standardized to the muscle weight is presented in the Appendix (Additional file 1: Additional Results and Additional file 1: Fig. S1).

### Muscle weight

There was a signifcant effect of immobilisation (*p* < 0.001), inflammation (*p* < 0.001) and their interaction immobilisation with inflammation (*p* = 0.024), as well as the side examined—ipsilateral versus contralateral (*p* < 0.001), with an interaction of immobilisation × side (*p* < 0.001), but not inflammation × side (*p* = 0.07) on muscle weight. There was no effect of insulin (*p* = 0.89) on muscle weight.

**Fig. 4:**
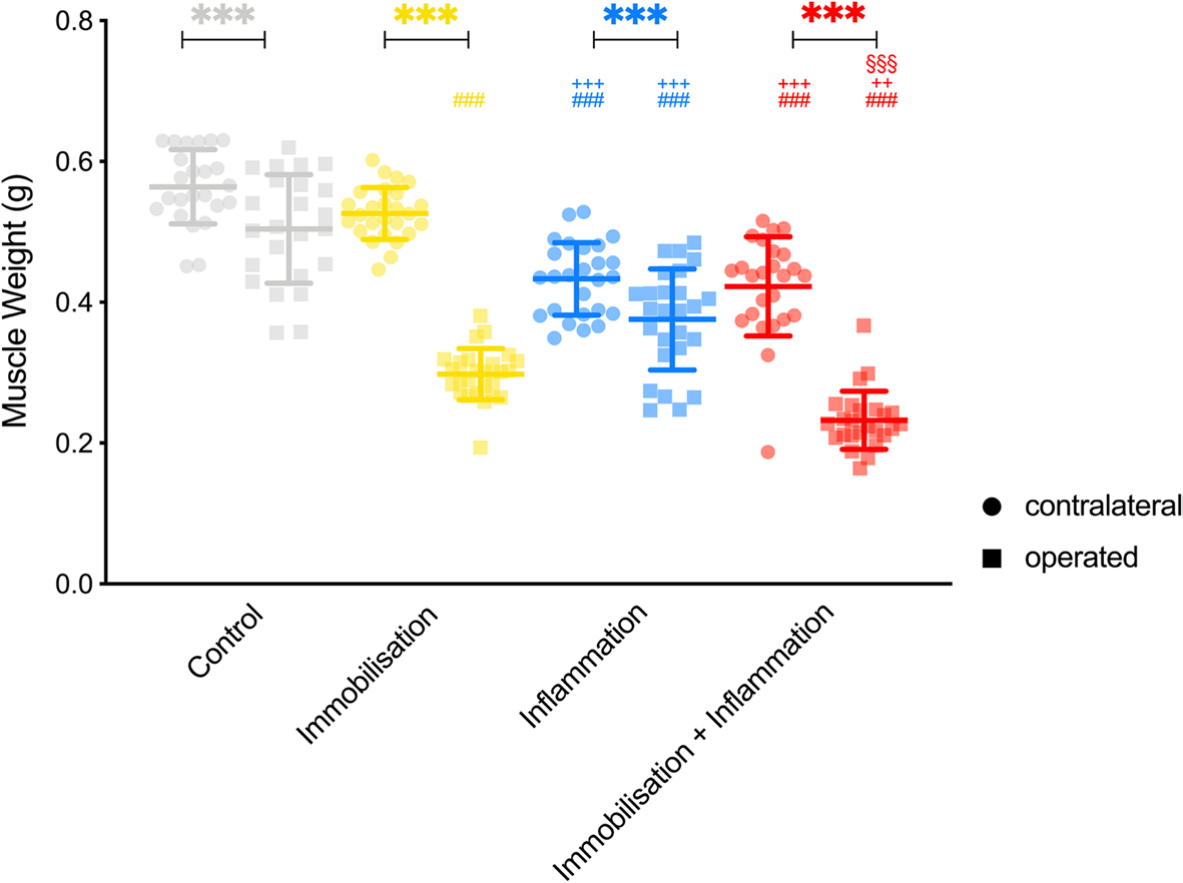
Muscle weight in gram. In all 4 groups a significant difference in muscle weight between the operated and contralateral leg was present (*p* < 0.001 for all four groups). The control group (mean [SD]; contralateral: 0.56 [0.05]; operated: 0.50 [0.08]) had significantly higher muscle weights in the contralateral and operated leg in comparison with the inflammation (mean [SD]; contralateral: 0.43 [0.05]; *p* < 0.001; operated: 0.38 [0.07]; *p* < 0.001) as well as immobilisation and inflammation group (mean [SD]; contralateral: 0.42 [0.07]; *p* < 0.001; operated: 0.23 [0.04]; *p* < 0.001). Meanwhile, only in the operated leg a significant difference between the control and immobilisation group (mean [SD]; operated: 0.30 [0.04]; *p* < 0.001) was present. The immobilisation group showed a significantly higher muscle weight in the contralateral leg (mean [SD]; contralateral: 0.53 [0.04]) in comparison with the inflammation (*p* < 0.001) as well as immobilisation and inflammation group (*p* < 0.001) while showing a lower muscle weight in the operated leg in comparison with the inflammation group (*p* < 0.001) but a higher muscle weight in the operated leg in comparison with the immobilisation and inflammation group (*p* = 0.001). When comparing the inflammation group to the immobilisation and inflammation group a significantly higher muscle weight in the operated leg was present (*p* < 0.001). The number of identical symbols at the top of the error bars indicate the significance level: one < 0.05; two < 0.01 and three < 0.001. *Significant difference between the operated and contralateral leg within the same group. ^#^Significant difference to the control group for the respective leg. ^+^Significant difference to the immobilisation group for the respective leg. ^§^Significant difference to the inflammation group for the respective leg

The effect of immobilisation alone was estimated to be − 0.10 g (95% CI − 0.12 to − 0.08), of inflammation − 0.11 g (95% CI − 0.13 to − 0.09) and of surgery − 0.13 g (95% CI − 0.15 to − 0.12) (Fig. 4).

### Phosphorylation of key enzymes of the insulin signaling pathway

#### Phosphorylated protein kinase b (pAkt)/total protein kinase b (Akt)

**Fig. 5:**
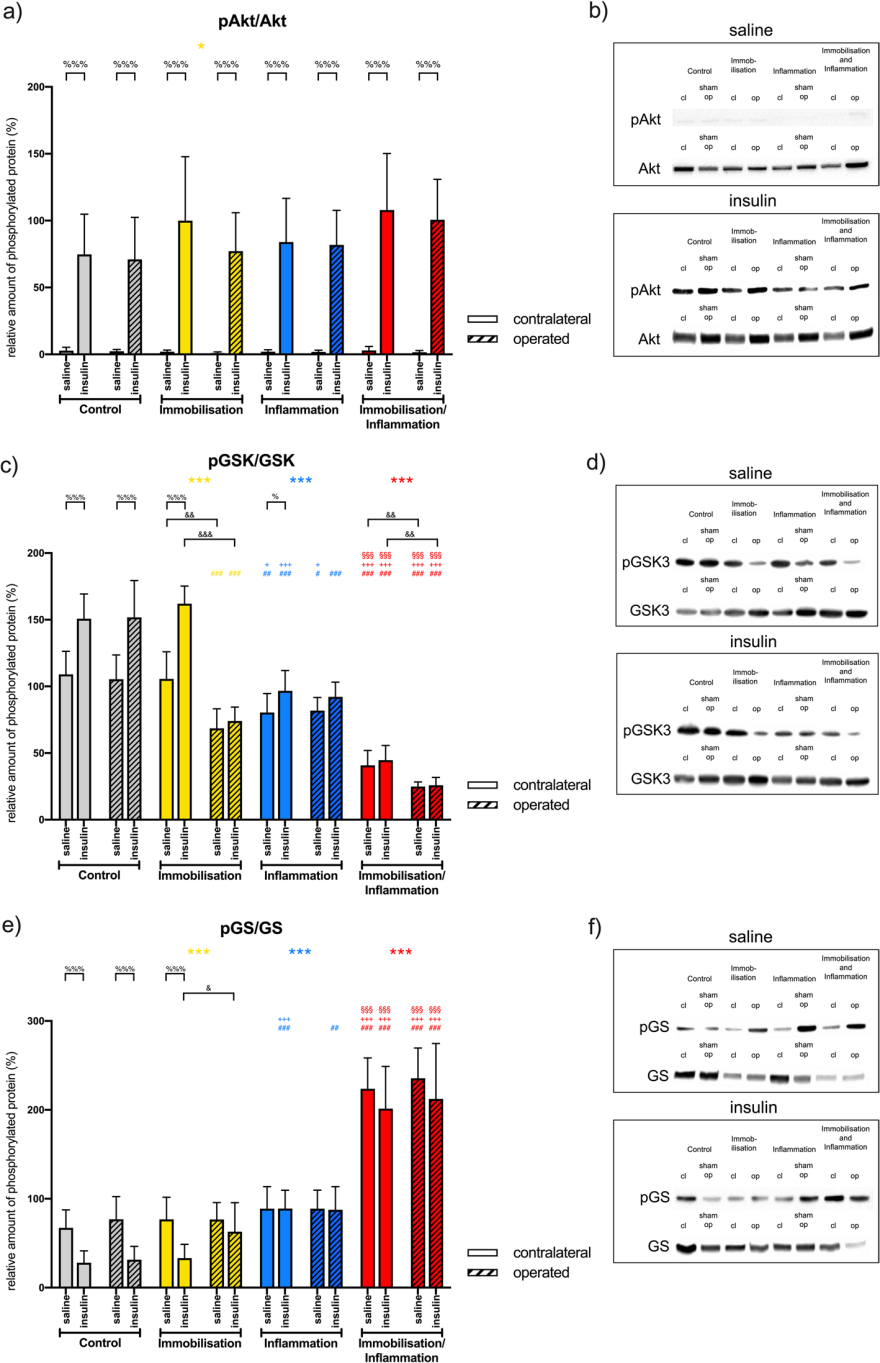
Relative amount as well as western blots of phosphorylated key enzymes of the insulin signaling pathway. The number of identical symbols at top of each box indicate the significance level: one < 0.05; two < 0.01 and three < 0.001. Expression values were normalised to total protein. Protein kinase b = Akt; phosphorylated Protein kinase B = pAkt; glycogen synthase kinase 3 beta (GSK3); phosphorylated glycogen synthase kinase 3 beta (pGSK3); Glycogen synthase = GS; phosphorylated Glycogen synthase (pGS); contralateral = cl; operated = op; sham-operated = sham op. *Significant the main effect of the factors immobilisation, inflammation as well as immobilisation and inflammation. ^#^Significant difference to the control group for the respective leg. ^+^Significant difference to the immobilisation group for the respective leg. ^§^Significant difference to the inflammation group for the respective leg. ^%^Significant difference of the insulin group to the saline group for the respective group. ^&^Significant difference of the operated leg to the contralateral leg for the respective group

**Fig. 6:**
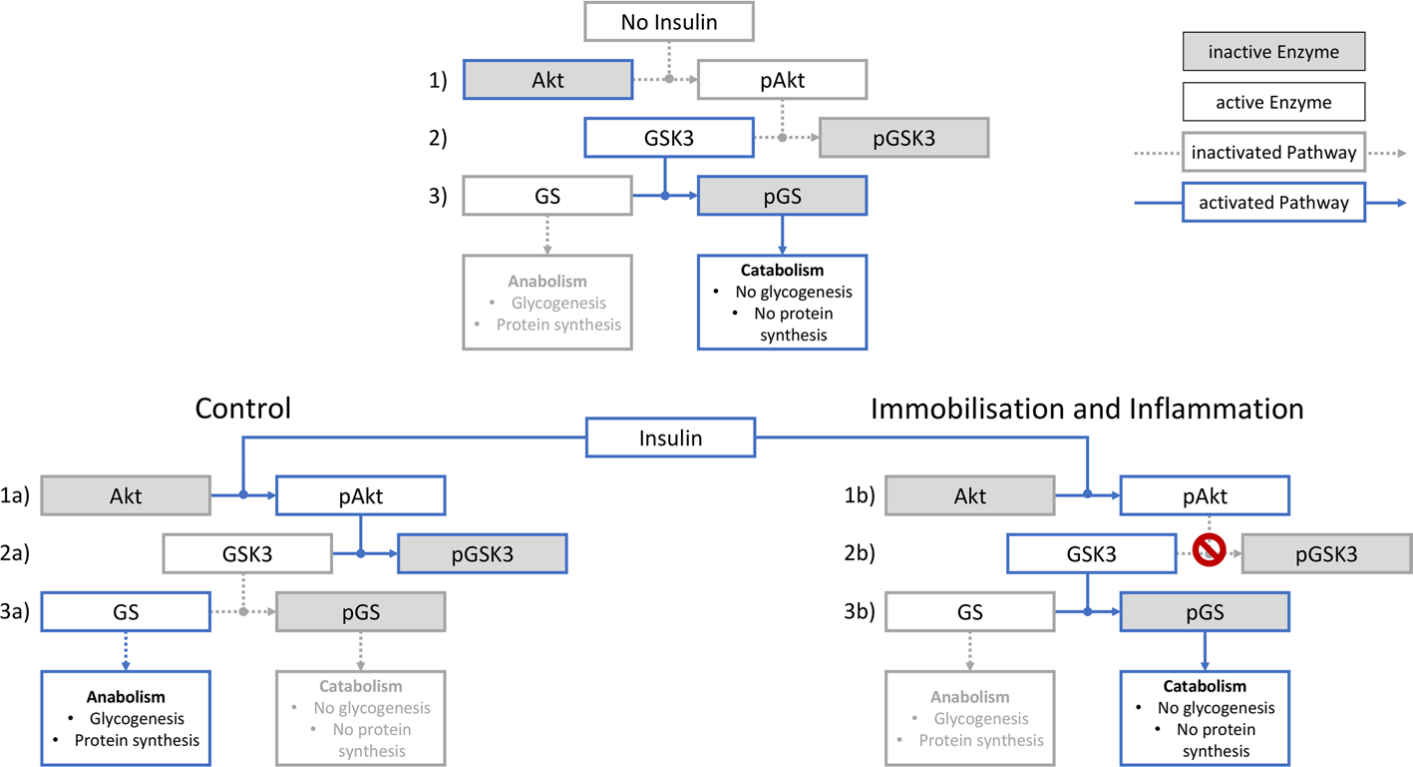
Impairment of the Akt–GSK3–GS pathway in skeletal muscle. No insulin (Control and intervention groups with saline): (1) Akt in its deactivated state. (2) No inhibition through Akt, hence GSK 3 remains activated. (3) This leads to deactivation of GS via phosphorylation. → Catabolism is enabled Control (Control group with insulin): (1a) Insulin leads to activation of Akt to phosphorylated Akt. (2a) This causes deactivation of GSK3 to pGSK3. (3a) Consequently, GS remains activated. → Anabolism is enabled. Immobilisation and Inflammation (Intervention group with insulin): (1b) Insulin leads to activation of Akt to pAkt. (2b) GSK3 remains activated as inhibition via pAkt is impaired. (3b) This results in deactivation of GS to pGS. → Catabolism is enabled

Immobilisation [estimated effect size (95% CI) 9.1-fold (0.2 to 17.9); *p* = 0.045] and insulin [estimated effect size (95% CI) 85.1-fold (76.2 to 94.0); *p* < 0.001] had an independent effect on Akt phosphorylation. Post-hoc, no differences between the groups could be observed irrespective of the administration of insulin or saline. Insulin led to a significant increase in Akt phosphorylation in all groups but did not significantly differ from each other (Figs. 5a, b, 6).

#### Phosphorylated glycogen synthase kinase 3 beta (pGSK3)/glycogen synthase kinase 3 beta (GSK3)

All main factors (immobilisation, inflammation, insulin, side) were highly significant (*p* < 0.001). Similarly, were the interaction terms immobilisation × side, inflammation × side, immobilisation × inflammation × side (*p* < 0.001) and insulin × side highly significant (*p* = 0.008). The estimated effect sizes were − 40.2 (95% CI − 45.6 to − 34.8) for immobilisation, − 55.0 (95% CI − 60.4 to − 49.5) for inflammation and 22.7 (95% CI 17.3 to 28.2) for insulin. These effects lead to significantly reduce amounts of pGSK3 in the immobilisation and inflammation group in comparison with all others groups. The additive nature of the two interventions is displayed in the signifcant reduction in the operated compared to the contralateral leg within the immobilisation and inflammation group. The control group on the other hand as well as the contralateral leg of the immobilisation and also the inflammation group showed a significant increase in pGSK3 after insulin stimulation (Figs. 5c, d, 6).

#### Phosphorylated glycogen synthase (pGS)/glycogen synthase (GS)

A highly significant (*p* < 0.001) effect for immobilisation {estimated effect size [95% CI 70.6-fold (58.8 to 82.4)]}, inflammation {estimated effect size [95% CI 96.7 (85.0 to 108.5)]} and insulin {estimated effect size [95% CI − 23.6 (− 35.4 to − 11.8)]} as main factors could be observed, while only the interaction term immobilisation × inflammation was significant (*p* < 0.001). The immobilisation with inflammation group showed significantly increased amounts of pGS in comparison with all other groups without a difference between the muscles. Furthermore, no response to insulin stimulation was evident. In contrast, the control group as well as the contralateral leg of the immobilisation group had a significant reduction of pGS with insulin stimulation (Figs. 5e, f, 6).

## Discussion

In a rat double-hit model combining two main risk factors for ICUAW (immobilisation and inflammation), we found a significant muscle atrophy as well as an impaired insulin signaling pathway. The insulin signaling pathway was intact up to the level of Akt phosphorylation. Although Akt was phosphorylated, the function of pAKT seemed to be impaired as the downstream effect on GSK3 phosphorylation was impaired resulting in an increased GS phosphorylation and its deactivation. None of these mechanisms showed a response to insulin stimulation indicating severe insulin resistance.

Immobilisation and inflammation lead to muscle atrophy, with an additive effect of both conditions combined. These findings are in line with previously published reports using the same model and demonstrate an effective ICUAW model [14, 15]. Furthermore, the persistent elevation in Methaemoglobin after injection of *Corynebacterium parvum* are indicative of a sufficient inflammation [15].

We also observed a decrease in muscle weight in the contralateral leg of the immobilisation group compared to controls as well as a significant decrease due to the sham operation in the intraindividual comparison, which can be interpreted as decreased mobility due to either the contralateral immobilisation or surgical pain.

The insulin signaling pathway showed significant alterations due to inflammation and immobilisation. Akt/pAkt is a key mediator in muscle atrophy and insulin resistance. Previous research has shown that overexpression of Akt leads to muscle hypertrophy and can prevent atrophy, while the Akt phosphorylation is downregulated during muscle atrophy [19–21]. Furthermore, it has been shown that a dysfunctional kinase activity of Akt reduced GLUT4 translocation by 87%, which resulted in severe insulin resistance similar to critically ill patients [5, 22, 23]. Activation of Akt via phosphorylation after insulin stimulation was unhampered in our study confirming recent findings in critically ill patients [5, 24]. This indicates that insulin signaling up to the point of Akt phosphorylation was intact and that the defect is likely downstream of pAkt. Nevertheless, there are conflicting results regarding pAkt in critically ill patients as well as animals, which show a downregulation of pAkt due to inflammation [5, 13, 25]. The exact role of pAkt/Akt still needs further investigation.

Regulation of muscle mass downstream of Akt is mediated via the kinase mTOR-s6 kinase-1 (s6K1) pathway. [19, 26, 27]. We focussed on GSK and observed a significant decrease in phosphorylation due to immobilisation and inflammation, which is in line with the findings in critically ill patients [25]. This decrease in phosphorylation leads to an insufficient deactivation after stimulation with insulin pointing towards an increased GSK3 activity during critical illness.

GSK3 has been established as relevant mediator in ubiquitin–proteasome-mediated muscle atrophy that also mediates atrophy during critical illness [28, 29]. Hence, inhibition of GSK3 leads to muscle sparing during models of critical illness [30]. The observed increase in active GSK3 with insufficient deactivation confirmed its involvement in muscle atrophy during critical illness and suggests that insulin resistance originates from insufficient phosphorylation via Akt.

GSK3 has been shown to inhibit Adenosine monophosphate-activated protein kinase (AMPK) as part of the anabolic insulin pathway [31]. AMPK initiates catabolic processes in energy depleted cells such as muscle atrophy through induction of muscle protein degradation as well as inhibition of muscle protein synthesis [32–34]. This helps replenish the energy stores by promoting glucose uptake via GLUT4 [35]. Insufficient AMPK deactivation reflected in an increased pAMPK/AMPK-ratio has been observed in critically illy patients and would also explain the phenotype of ICUAW with muscle atrophy [35, 36]. Inhibition of AMPK via GSK3 would, therefore, be a plausible mechanism. On the other hand would an increased activation of AMPK potentially reverse the insufficient GLUT4 translocation and, therefore, improve glucose uptake into skeletal muscle as it has also been evidenced to be disrupted during critical illness [5].

Active GSK3 prevents dephosphorylation and activation of GS leading to insufficient glycogen synthesis, which is confirmed within our data as we observed a significantly increased content of pGS in animals submitted to immobilisation and inflammation while showing no response to insulin stimulation [37]. A low muscle glycogen has been shown to correlate with fatiguability measured as exercise time until exhaustion [38]. Our results of insufficient GSK3 phosphorylation and the consecutive phosphorylation and deactivation of GS propose a mechanism for the low muscle glycogen content that has been observed in the muscle of critically ill patients [39]. This could explain recent observations showing that even though a regain of muscle mass can be observed after critical illness, patients still suffer from impaired muscle function confirming that ICUAW extends beyond muscle atrophy [40]. Depletion of glycogen as the main fuel source would be one explanation for fatigue and muscle dysfunction [41].

While our investigation adds new insights to the pathophysiology of ICUAW there are, of course, certain limitations. First, the results stem from an animal model that cannot account for all risk factors present during an intensive care therapy and, therefore, need to be confirmed in a translational approach in the future. Moreover, there are multiple pathways involved in synthesis and degradation of muscle as well as in the glucose homeostasis, respectively, insulin resistance, which were not all investigated in this study.

## Conclusion

Immobilisation and inflammation lead to an impairment in the insulin signaling pathway downstream of Akt causing an insufficient phosphorylation and deactivation of GSK after an insulin stimulus. This could directly contribute to muscle atrophy, and furthermore, phosphorylated and deactivated GS could hamper glycogen disposition and promote muscle fatigue.

## Supplementary Information

**Additional file 1:** Appendix.

## Data Availability

The data sets used and/or analysed during the current study are available from the corresponding author on reasonable request.

## Abbreviations

ICUAW: Intensive care unit acquired weakness
ICU: Intensive care unit
Akt: Protein kinase b
pAkt: Phosphorylated protein kinase b
GSK: Glycogensynthase-kinase-3-beta
pGSK: Phosphorylated glycogensynthase-kinase-3-beta
GS: Glycogen synthase
pGS: Phosphorylated glycogen synthase
MetHb: Methaemoglobin
