# Supplementary material for: Insulin signaling in skeletal muscle during inflammation and/or immobilisation
Source: Intensive Care Med Exp. 2023 Mar 27;11:16. doi: 10.1186/s40635-023-00503-9 (PMC10040391; doi:10.1186/s40635-023-00503-9)
Supplement: Supplementary file 1 — Additional file 1: Appendix. [file 40635_2023_503_MOESM1_ESM.pdf]

Appendix

Insulin Signaling in Skeletal Muscle During Inflammation and/or Immobilisation

Authors:

Julius J Grunow[1] MD, Thomas Gan[2] MD, Heidrun Lewald MD[2], J A Jeevendra Martyn[3] MD, FRCA, FCCM, Manfred Blobner[2] MD, Stefan J Schaller[2,1] MD

Affiliations:

1. Charité - Universitätsmedizin Berlin, corporate member of Freie Universität Berlin and Humboldt-Universität zu Berlin, Department of Anesthesiology and Operative Intensive Care Medicine (CCM/CVK), Charitéplatz 1, 10117 Berlin, Berlin, Germany
2. Technical University of Munich, School of Medicine, Klinikum rechts der Isar, Department of Anesthesiology and Intensive Care, Ismainger Straße 22, 81675 Munich, Bavaria, Germany
3. Department of Anaesthesia, Critical Care and Pain Medicine, Massachusetts General Hospital, Shriners Hospitals for Children®-Boston, and Harvard Medical School, 55 Fruit Street Boston, MA, USA

Corresponding author:

Univ.-Prof. Dr. Stefan J Schaller, MHBA

Department of Anesthesia and Intensive Care

Klinikum rechts der Isar, School of Medicine, Technical University of Munich

Ismaningerstr. 22

81675 Munich

s.schaller@tum.de

Table of Contents

Detailed Methods 3

Table A1. Antibodies for Western Blot 7

Additional Results: Body weight per muscle weight 8

Figure A1. Body weight per Muscle weight in gram per gram 8

References 9

Detailed Methods

Sample size

Sample size calculation was based on (p)Akt concentrations. Sugita et al. [1] were able to show a reduction within a burn model to 53% and a standard error of the mean of 7% and 6%, resulting in standard deviations of 22.1% and 15.8%, respectively. We set a 25% reduction of the p-Akt/PKB activation as relevant difference to show insulin resistance. This led to a critical difference

$$D = \frac{25\%}{\sqrt{\frac{\left(7\% * \sqrt{10}\right)^2 + \left(5\% * \sqrt{10}\right)^2}{2}}} = 1.21$$

We set an alpha level of 0.003125 after correction for multiple testing (0.05/16 = 0.003125) with 16 different groups (8 different interventional groups and 2 legs). A beta value of 0.2 within a one-sided t-test leads to necessary number of legs of 20, i.e., 10 animals per treatment group. According to previous studies using this model we expected a drop-out rates of 15% due to the study procedures (e.g., surgery and anaesthesia), 10% due to insufficient immobilisation and 35% due to severity of inflammation and animal protection in the respective groups (e.g., 35% are only calculated in inflammation groups). We therefore arrive at a sample size of 132 animals of which 52 are reserves animals.

General housing

Rats were acclimatized to standard conditions of the animal laboratory for at least seven days. Animal housing facility was subject to a person-limited access control, air conditioning and a light-dark rhythm of 12 hours each. Animals were kept in a pathogen-free environment in macrolon cages type III and IV. Food and drinking water

were available ad libitum. Body weight of animals was recorded, and their clinical condition was evaluated daily.

Anaesthesia

Animals were put under general anaesthesia with isoflurane on day 0 and then intubated and ventilated with 14 Gauge intravenous catheters (B. Braun Melsungen, Germany). For analgesia, 0.02 mg/kg buprenorphine was injected. Subsequent monitoring included monitoring of vital signs and ventilation by continuous end expiratory CO2 measurements.

Immobilization

Immobilization was achieved by fixation of knee and ankle joint of one hindlimb at a 90-degree angle using Kirschner wires of 1.0 mm diameter. The contralateral leg served as control.

Inflammation

Animals received injections of 56 mg/kg heat-inactivated Corynebacterium parvum (Hoffmann-La Roche, Switzerland) via the dorsal vein of the penis on day 0 under isoflurane anaesthesia. To maintain systemic inflammation over the entire course of the experiment, repetitive injections were given on days 4 and 8. For this, a short isoflurane anaesthesia was induced via glass chamber and then maintained via nasal mask.

Inflammation was monitored by daily body weight measurements and venous methemoglobin controls on days 0, 4, 8 and 12. For blood collection and Corynebacterium parvum injections a venous catheter size 26G (B. Braun Melsungen,

Germany) was used. Animals in sham inflammation groups received 0.5 ml saline instead of Corynebacterium parvum. Otherwise, the procedure was identical.

Insulin injection

On day 12, animals were put under general anaesthesia after at least six hours of fasting (water unrestricted). Anaesthesia, analgesia, intubation, ventilation and monitoring were equivalent to procedures for immobilization. Fasting before insulin stimulation was important to generate fasting blood glucose levels for the experiment. Then, the abdominal cavity was opened under deep isoflurane anaesthesia. Subsequently, insulin groups received 0.65 IU/kg insulin injected via the portal vein. Five minutes later, tibialis muscles of both legs were harvested, weighed, immediately frozen in liquid nitrogen and stored at -80°C for western blot analysis. Animals were then euthanized under deep general anaesthesia. Animals of sham insulin groups received saline instead of insulin. Otherwise, the procedure was identical.

Western Blot

Therefore, tibialis muscle was homogenized in RIPA lysis buffer containing protease inhibitor, using a tissue lyser (TissueRuptor II, Qiagen, Germany). Samples were centrifuged and aliquots of the supernatant containing equal amounts of protein by Bio-Rad Protein Assay Kit II (Bio-Rad Laboratories, USA) were subjected to TGX StainFree™ Gels (Bio-Rad Laboratories, Munich, Germany). Afterwards immunoblotting was performed. Equal amounts of protein (40µg) per lane were subjected to SDS-PAGE and then blotted onto Amersham™ Hybond 0.2µm PVDF Low Fluorescence membrane (GE Healthcare, Life Sciences, USA). Membranes were blocked by Roti®-Block (Carl Roth GmbH + Co. KG, Karlsruhe, Germany) and

incubated in primary antibody at 4° Celsius for at least 16 hours. Primary antibodies included (Table A1): Akt antibody, Phospho-Akt (Ser473) (D9E) Rabbit monoclonal antibody (mAb), GSK-3β (D5C5Z) XP Rabbit mAb, Phospho-GSK-3β (Ser9) (D85E12) XP Rabbit mAb, Glycogen Synthase Antibody, Phospho-Glycogen Synthase (Ser641) Antibody (all dilution 1:5000, Cell Signaling Technology, USA). Then, membranes were incubated in secondary anti-rabbit IgG, HRP-linked antibody (Cell Signaling Technology, USA) for 60 min (dilution 1:10.000). Marked proteins were detected by Bio-Rad Molecular Imager® ChemiDoc™ XRS+ (Bio-Rad Laboratories GmbH, Munich, Germany) and analysed with ImageLab (Bio-Rad Laboratories GmbH, Munich, Germany) using total protein normalization with stain-free technology [2]. Evaluation of the images was performed via ImageLab (Bio-Rad Laboratories GmBH, Munich, Germany),

Table A2. Antibodies for Western Blot

Protein	Name of Antibody	Company
Akt	Akt Antibody	Cell Signaling Technology, USA
Phosphorylated Akt (pAkt)	Phospho-Akt (Ser473) (D9E) XP Rabbit mAb	Cell Signaling Technology, USA
Glycogensynthase-Kinase 3 (GSK3)	GSK-3β (D5C5Z) XP Rabbit mAb	Cell Signaling Technology, USA
Phosphorylated Glycogensynthase-Kinase 3 (pGSK3)	Phospho-GSK-3β (Ser9) (D85E12) XP Rabbit mAb	Cell Signaling Technology, USA
Glycogensynthase (GS)	Glycogen Synthase Antibody	Cell Signaling Technology, USA
Phosphorylated Glycogensynthase (pGS)	Phospho-Glycogen Synthase (Ser641) Antibody	Cell Signaling Technology, USA
**Secondary Antibody**	Anti-rabbit IgG, HRP-linked Antibody	Cell Signaling Technology, USA

Additional Results: *Body weight per muscle weight*

The main factors immobilisation, inflammation and side ($p < 0.001$) had a significant effect. Furthermore, the interaction of side x immobilisation had a significant effect ($p < 0.001$).

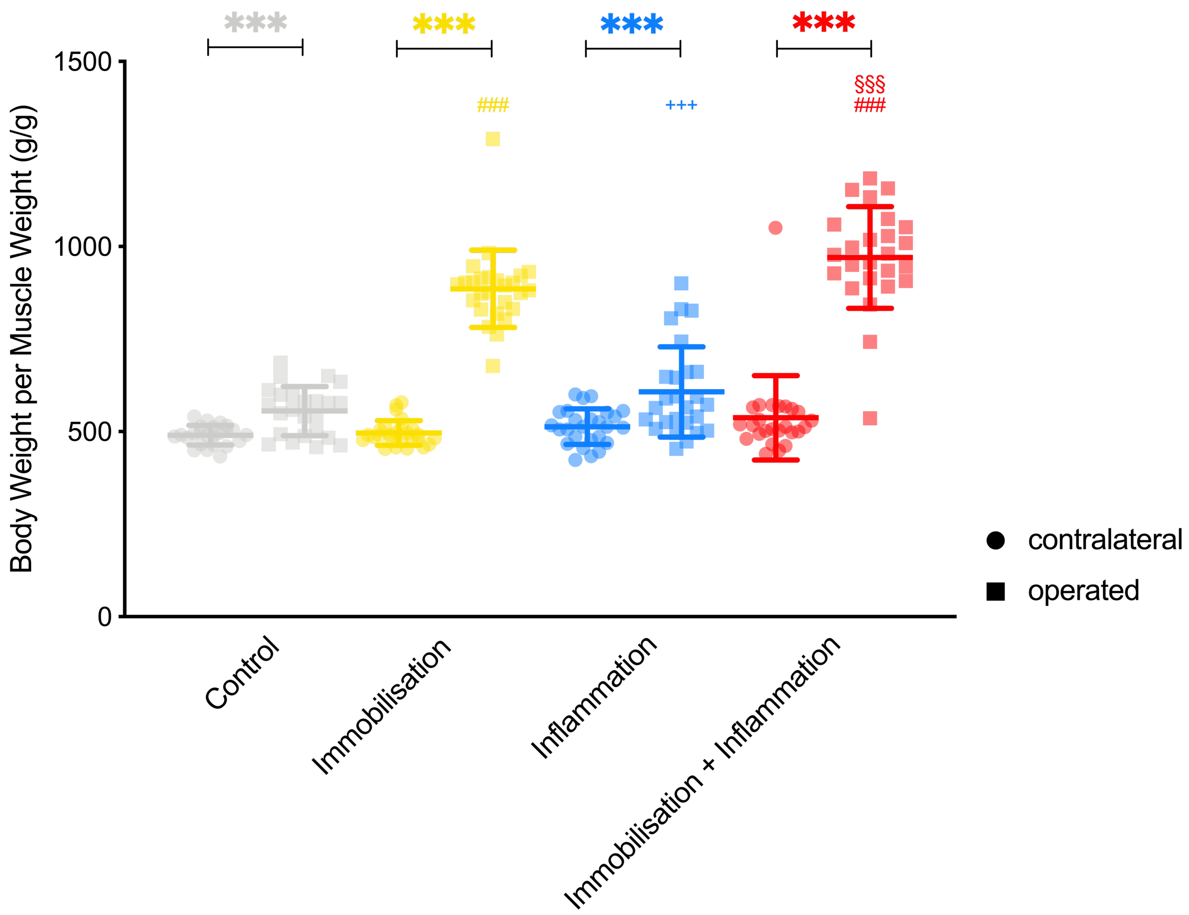

Fig. A1 Body weight per Muscle weight in gram per gram
In all 4 groups a significant difference between the operated and contralateral leg was present ($p < 0.001$ for all four groups).
The control group (mean [SD]; contralateral: 490.65 [26.74]; operated: 556.00 [66.09]) and the inflammation group (mean [SD]; contralateral: 513.58 [48.08]; $p < 0.001$; operated: 607.65 [122.09]) had a significantly lower body weight to muscle weight ratio than the immobilisation (mean [SD]; contralateral: 496.70 [33.43]; mean [SD]; operated: 885.94 [104.51]; $p < 0.001$ for both comparison) and immobilisation and inflammation (mean [SD]; contralateral: 537.74 [113.80]; $p < 0.001$; operated: 970.85 [137.65]; $p < 0.001$ for both comparison) group in the operated leg.
The number of identical symbols at the top of the error bars indicate the significance level: one < 0.05; two < 0.01 and three < 0.001.

∗ showed significant difference between the operated and contralateral leg within the same group
# showed significant difference to the control group for the respective leg
+ showed significant difference to the immobilisation group for the respective leg
§ showed significant difference to the inflammation group for the respective leg

References

1. Sugita H, Kaneki M, Sugita M, Yasukawa T, Yasuhara S, Martyn JA, (2005) Burn injury impairs insulin-stimulated Akt/PKB activation in skeletal muscle. Am J Physiol Endocrinol Metab 288: E585-591
2. Gurtler A, Kunz N, Gomolka M, Hornhardt S, Friedl AA, McDonald K, Kohn JE, Posch A, (2013) Stain-Free technology as a normalization tool in Western blot analysis. Anal Biochem 433: 105-111
